# Modulation of Synaptic Plasticity by Vibratory Training in Young and Old Mice

**DOI:** 10.3390/brainsci11010082

**Published:** 2021-01-10

**Authors:** Ida Cariati, Roberto Bonanni, Gabriele Pallone, Giuseppe Annino, Virginia Tancredi, Giovanna D’Arcangelo

**Affiliations:** 1 Department of Clinical Sciences and Translational Medicine, “Tor Vergata” University of Rome, Via Montpellier 1, 00133 Rome, Italy; ida.cariati@uniroma2.it; 2 Department of Systems Medicine, “Tor Vergata” University of Rome, Via Montpellier 1, 00133 Rome, Italy; roberto.bonanni1288@gmail.com (R.B.); gabriele.pallone@gmail.com (G.P.); g_annino@hotmail.com (G.A.); giovanna.darcangelo@uniroma2.it (G.D.); 3 Centre of Space Bio-Medicine, “Tor Vergata” University of Rome, Via Montpellier 1, 00133 Rome, Italy

**Keywords:** vibratory training, synaptic plasticity, hippocampus, aging, whole body vibration

## Abstract

In the past 40 years, scientific research has shown how Whole Body Vibration concept represents a strong stimulus for the whole organism. Low (<30 Hz), medium (30–80 Hz), and high (>80 Hz) frequency vibrations can have both positive and negative effects, depending on the oscillation type and duration of exposure to which the body is subjected. However, very little is known about the effects of vibratory training on the brain. In this regard, we verified whether three vibratory training protocols, differing in terms of vibration frequency and exposure time to vibration, could modulate synaptic plasticity in an experimental mouse model, by extracellular recordings in vitro in hippocampal slices of mice of 4 and 24 months old. Our results showed that vibratory training can modulate synaptic plasticity differently, depending on the protocol used, and that the best effects are related to the training protocol characterized by a low vibration frequency and a longer recovery time. Future studies will aim to understand the brain responses to various types of vibratory training and to explore the underlying mechanisms, also evaluating the presence of any structural and functional changes due to vibratory training.

## 1. Introduction

Many studies on vibrations published in the previous century have focused on the harmful effects of mechanical vibrations in the workplace [1]. It has been shown that exposure to these vibration levels mainly leads to an increase in the health risks of the musculoskeletal system and the peripheral nervous system [2,3]. However, more recent studies have found positive effects of Whole Body Vibration (WBV) experimentally and/or therapeutically induced, suggesting that WBV represents a safe and effective way to train the musculoskeletal system and can be clinically applied to sports medicine. It has been observed that WBV improves the physiological and health-related components of physical fitness, such as higher bone density [4,5] and lower blood pressure [6]. An increase in muscle strength [7,8,9] and a reduction in knee osteoarthritis symptoms [10] have also been reported. Additionally, WBV has been recommended as an effective alternative strength training in the elderly, as it would appear to improve mobility, balance, and general health [11,12,13], as well as body composition, insulin resistance, and glucose regulation [14]. The application of mechanical vibrations to the human body is also capable of producing an adaptive hormonal response [15], inducing an increase in the plasma concentration of testosterone and growth hormone (GH), and a decrease in the concentration of cortisol. Vibration also stimulates the production of neurotrophins, a family of proteins that act by regulating the natural cell death of neurons that occurs during development; moreover, they can stimulate the survival of distinct populations of neurons in vitro [16]. Not surprisingly, WBV is currently also considered a complementary training to standard physical rehabilitation programs, including those related to the treatment of neurodegenerative diseases [17,18,19]; however, preclinical research is needed to reveal the underlying brain mechanism. Indeed, not only the optimal frequency and amplitude need to be identified, but also the level of muscle activation that would benefit more from vibration stimulation [20]. Therefore, considering the many possible combinations of amplitudes and frequencies that can be achieved with current technology, there are a wide variety of WBV protocols that could be used on humans for therapeutic purposes.

Despite numerous evidence about the beneficial effects of WBV on the human body, very little is known about the effects of vibratory training on the brain. It is assumed that WBV induces sensory stimulation in the cerebral cortical regions through the activation of skin, tendon and muscle receptors that respond to vibrations [21,22,23,24]. The vibratory sensations transmit via the dorsal column of the spinal cord to the primary somatosensory area of cortex, through vibratory receptors whose purpose is to make us aware of the different frequencies of external stimuli. Since the vibratory stimulus is perceived and conducted to the brain via the integumentary and nervous system, any factor that interrupts the neuronal transmission from the vibratory receptors in the deep dermis to the somatosensory area of the cortex affects the vibratory sensations. In this regard, it is known that altered vibration perception and the pathways involved are linked to the onset of spinal diseases, which manifest themselves in loss of position, vibration, and tactile sensations [25]. To date, it is not known whether this process of vibratory stimuli transmission can also affect the structures present in the limbic system, for example the hippocampus, which plays a fundamental role in mnemonic processes.

Based on this evidence, the aim of our work was to verify whether vibratory training can modulate synaptic plasticity in an experimental mouse model, evaluating the presence of any functional changes by extracellular recordings in vitro in mouse hippocampal slices. In this regard, we applied three different vibratory training protocols, to assess whether any effects observed at the hippocampal level could depend on the use of different training protocols in terms of vibration frequency and exposure time to vibration. Finally, since it is known that synaptic plasticity is closely related to age [26,27], in our experimental work we used mouse models of two different age groups, 4 months and 24 months, comparing the results obtained with those of the respective control groups who have not performed any type of vibratory training.

## 2. Materials and Methods

### 2.1. Animals

Wild type BALB/c male mice were used in accordance with guidelines and regulations of the European Union Council Directive (86/609/European Economic Community). All the experimental protocols were approved by the Italian Ministry of Public Health (authorization n. 86/2018-PR). We used a total of 32 mice, divided into groups based on age and vibratory training protocol. Specifically, the 4-month-old mice were divided into five groups: three groups of trained mice (4 mice per group), each one submitted to different vibratory training protocol, and two control groups (4 mice per group). As for the 24-month-old mice, they were divided into three groups (4 mice per group): a trained group and two control groups. For both age groups, one control group (CTRL SED) included sedentary mice not subjected to any type of training; while the other control group (CTRL WBV) included mice subjected to the same regimen of placement on the box in the platform, the same environmental exposure including motor sounds, but are not subjected to vibratory training. The health status of animals was monitored daily by resident veterinarians and experimenters, who also measured animal weight during the training period. All experimental animals were kept under the same housing conditions and diet.

### 2.2. WBV Training Protocol

**Figure 1:**
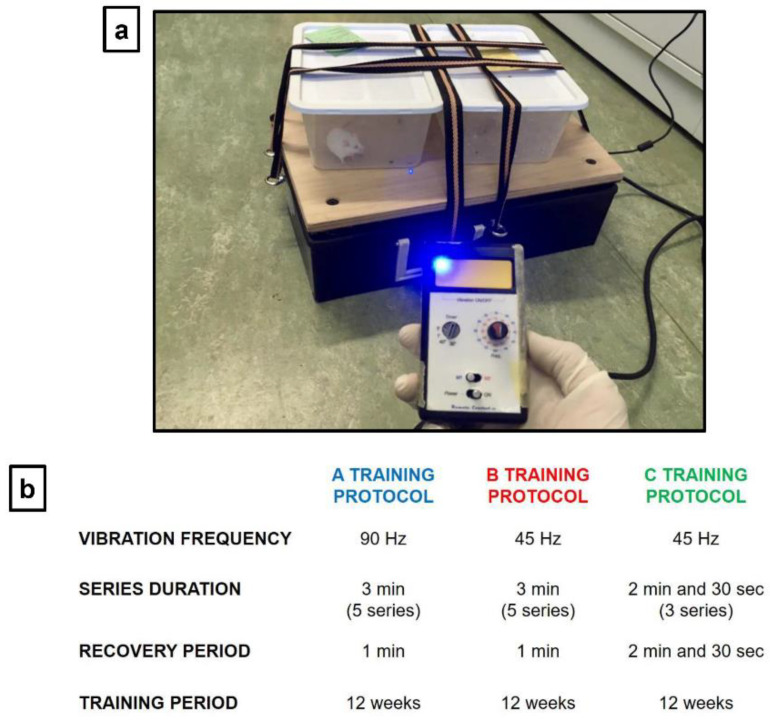
(**a**) A vibrating platform (Power Club, Vigarano Mainarda, 44049 FE, Italy) was used to subject animals to Whole Body Vibration (WBV). It is characterized by a power supply of 220 V and a total maximum electrical power of 0.12 kW. The function generator generates a sinusoidal control signal at the frequencies used (45 Hz and 90 Hz). (**b**) Vibratory training was conducted using three protocols (A, B and C), which differ in terms of vibration frequency and exposure time to vibration. A and B protocols consisted of 5 series (consecutive number of repetitions) of 3 min each, interspersed with 1 min of recovery. The C protocol consisted of 3 series of 2 min and 30 s each, with a recovery period of equal duration. The vibration frequency used was 90 Hz for the A protocol and 45 Hz for B and C protocols.

Each experimental group underwent vibratory training sessions using a vibrating platform (Power Club, Vigarano Mainarda, 44049 FE, Italy) (Figure 1a). It is characterized by a power supply of 220 V, a total maximum electrical power of 0.12 KW, a minimum vibration frequency of 45 Hz and a maximum vibration frequency of 90 Hz. In particularly, at a vibration frequency of 45 Hz, we recorded an acceleration of 2 g and a shift of 1.5 mm; while, at a vibration frequency of 90 Hz, we recorded an acceleration of 2.8 g and a shift of 1.1 mm. 

Vibratory training was conducted using three protocols (A, B, and C), which differ in terms of vibration frequency and exposure time to vibration (Figure 1b). A and B training protocols are WBV protocols, characterized by 5 series (consecutive number of repetitions), each lasting 3 min, with 1-min recovery between one series and other. They differ only in the vibration frequency used, which is 90 Hz for the A protocol and 45 Hz for the B protocol. On the other hand, the C protocol is characterized by 3 series, each lasting 2 min and 30 s, with a recovery period of the same duration (2 min and 30 s); as for as the B protocol, the vibration frequency used is 45 Hz. A and B protocols were designed to assess whether exposure to two different vibration frequencies could induce changes in synaptic plasticity in young mice. At the same time, to evaluate these changes in old mice, we designed the C protocol, which being characterized by a longer recovery period and a lower number of series, could be the most suitable for this delicate age group. The training was conducted for a total of 12 weeks, with a three-weekly frequency, therefore, each group of mice carried out 36 days of activity. Specifically, the animals were raised on a light: dark cycle of 12: 12 h, and training was carried out in the morning, between 10 and 11 am.

### 2.3. Extracellular Recordings in Mouse Hippocampal Slices

The animals belonging to the different experimental groups were sacrificed after the 12 weeks of training as well as the sedentary animals. All efforts were made to minimize the number of animals used and their suffering. Under anesthesia with halothane (2-Brom-2-chlor-1,1,1-trifluor-ethan), the animals were decapitated, and brains were quickly removed and placed in cold, oxygenated artificial cerebral spinal fluid (ACSF), whose composition in mM was: NaCl 124, KCl 2, KH_2_PO_4_ 1.25, MgSO_4_ 2, CaCl_2_ 2, NaHCO_3_ 26, and Glucose 10. The hippocampal slices were prepared according to conventional procedures [28]. The hippocampus was rapidly dissected, and slices (450 µm thick) were cut transversely by a chopper (McIlwain Tissue Chopper) and transferred into an interface tissue chamber constantly perfused by a flow of 1.2 mL/min of ACSF and humidified gas (95% O_2_, 5% CO_2_) at 32–34 °C (pH 7.4). Extracellular recordings of the population spikes (PSs) were made in the stratum pyramidale of the CA1 subfield, with glass microelectrodes filled with 2 M NaCl (resistance 5–10 MΩ). Orthodromic stimuli (10–500 mA, 20–90 ms, 0.1 Hz) were delivered through a platinum electrode placed in the stratum radiatum in the Schaffer collateral/commissural CA1 pathways. The test stimulus intensity of 50-ms square pulses was adjusted to give a PS of 2–4 mV at 0.03 Hz. The PS amplitude, measured every time, corresponds to an average of 6 recordings/min. After recording stable signals (15–20 min), a tetanic stimulation (100 Hz, 1 s) was delivered to induce the Long-term Potentiation (LTP) at the same stimulus intensity used for the baseline responses. Signals were acquired, digitized, and stored using a personal computer with standard acquisition software (Axon, Foster City, CA, USA). Signal was fed to a computer interface (Digidata 1440A, Axon Instruments, Foster City, CA, USA) for subsequent analysis with the software PCLAMP10 (Axon Instruments, Foster City, CA, USA).

### 2.4. Statistical Analysis

Statistical analysis was performed using GraphPad Prism 8 Software (Prism 8.0.1, La Jolla, CA, USA). For electrophysiological experiments, data were expressed as mean ± SEM and *n* represent the number of slices analyzed. Data were compared with Two-way ANOVA and Tukey’s Multiple Comparison Test and were considered significantly different if *p* < 0.05.

## 3. Results

### 3.1. Effects of Vibratory Training on Body Weight in Young and Old Mice

**Figure 2:**
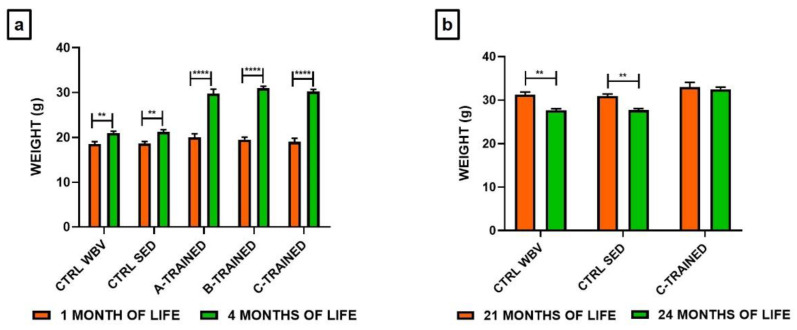
Effects of different training protocols on body weight. (**a**) Weight variations in young mice at 1 and 4 months of life were: CTRL WBV 18.5 ± 0.3 g vs. 21.0 ± 0.4 g (** *p* < 0.01); CTRL SED 18.6 ± 0.2 g vs. 21.3 ± 0.3 g (** *p* < 0.01); A-TRAINED 20.0 ± 0.8 g vs. 29.8 ± 1.0 g (**** *p* < 0.0001); B-TRAINED 19.5 ± 0.3 g vs. 31.0 ± 0.4 g (**** *p* < 0.0001); C-TRAINED 19.0 ± 0.4 g vs. 30.3 ± 0.5 g (**** *p* < 0.0001). (**b**) Weight variations in old mice at 21 and 24 months of life were: CTRL WBV 31.3 ± 0.3 g vs. 27.6 ± 0.3 g (** *p* < 0.01); CTRL SED 30.9 ± 0.2 g vs. 27.7 ± 0.3 g (** *p* < 0.01); C-TRAINED 33.0 ± 1.1 g vs. 32.5 ± 0.3 g.

We monitored the mice weight during the entire experimental period to verify if vibratory training had any effect on it. Figure 2 shows the comparison between the mean body weight values at the beginning and at the end of the training session. Regardless of the protocol used, we observed that vibratory training induced a significant increase in body weight in young mice (Figure 2a) (**** *p* < 0.0001). In fact, mean body weight prior to initiation of the training sessions was 20.0 ± 0.8 g in the A-TRAINED group, 19.5 ± 0.3 g in the B-TRAINED group and 19.0 ± 0.4 g in the C-TRAINED group. At the age of 4 months, after training sessions were completed, the mean body weight was 29.8 ± 1.0 g in the A-TRAINED group, 31.0 ± 0.4 g in the B-TRAINED group and 30.3 ± 0.5 g in the C-TRAINED group. For the control groups, instead, we observed an increase in body weight of only about 14% in both cases, with mean body weight values of 18.5 ± 0.3 g (CTRL WBV) and 18.6 ± 0.2 g (CTRL SED) for mice at 1 month of life and 21.0 ± 0.4 g (CTRL WBV) and 21.3 ± 0.3 g (CTRL SED) for mice at 4 months of life (** *p* < 0.01). 

Contrary to the results obtained for the young mice, we observed a slight decrease in body weight in old C-TRAINED mice at the end of the training period (Figure 2b), which however, is not statistically significant (at 21 months of life 33.0 ± 1.1 g, at 24 months of life 32.5 ± 0.3 g). Conversely for the control groups, we observed a significant decrease in body weight of about 10% in both cases, with mean body weight values of 31.3 ± 0.3 g (CTRL WBV) and 30.9 ± 0.2 g (CTRL SED) for mice at 21 months of life and 27.6 ± 0.3 g (CTRL WBV) and 27.7 ± 0.3 g (CTRL SED) for mice at 24 months of life (** *p* = 0.01).

### 3.2. Synaptic Plasticity Following Vibratory Training

**Figure 3:**
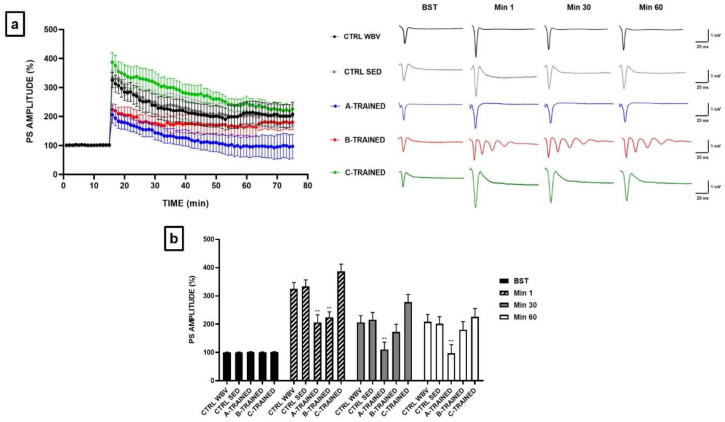
Synaptic plasticity in CA1 hippocampal subfield of young mice. (**a**) %PS amplitude as a function of time after HFS, applied at time t = 16, is shown in CTRL WBV (black line, *n* = 6), in CTRL SED (grey line, *n* = 6), in A-TRAINED (blue line, *n* = 6), in B-TRAINED (red line, *n* = 5), and in C-TRAINED (green line, *n* = 6) mice slices. The insert shows representative recordings obtained from slices of each experimental group. The first curve of each group refers to the BST and it was recorded before the application of a high-frequency stimulation (HFS, 100 Hz for 1 s), while the other curves refer to population spikes at times 1, 30 and 60 min after the HFS. (**b**) The PS values of BST (black bar), at min 1 (diagonal pattern bar), immediately after HFS, at min 30 (grey bar) and at min 60 (white bar) from the HFS are shown for each experimental group. Bars in the plot are means ± SEM of values obtained from different slices. Note that a significant statistical difference was reported between trained and control groups at min 1 (CTRL WBV and CTRL SED vs. A-TRAINED, ** *p* < 0.01; CTRL WBV and CTRL SED vs. B-TRAINED, ** *p* < 0.01), at min 30 (CTRL WBV and CTRL SED vs. A-TRAINED, ** *p* < 0.01) and at min 60 (CTRL WBV and CTRL SED vs. A-TRAINED, ** *p* < 0.01). The hippocampal slices (450 μm thick) were constantly perfused by a flow of 1.2 mL/min of ACSF and humidified gas (95% O_2_, 5% CO_2_) at 32–34 °C (pH 7.4).

The effects of different vibratory training protocols on the synaptic plasticity expression were analyzed in the CA1 region of hippocampal slices of young and old mice. Figure 3a shows how synaptic plasticity was modulated in a different way according to the type of vibratory training protocol performed by young mice. In particular, only the C protocol seemed to exert positive effects on synaptic plasticity, since we observed a significant increase in PS amplitude following HFS compared to the other experimental groups. Regarding the A and B protocols, we observed that the PS amplitude was significantly reduced in the first twenty min after HFS in both experimental groups compared to the control groups (** *p* < 0.01). In the remaining recording time, the PS amplitude did not seem to be affected in the B-TRAINED group; on the contrary, for the A-TRAINED group, it was considerably reduced, with statistically significant values compared to the other experimental groups (** *p* < 0.01). Furthermore, as shown in the insert of Figure 3a comparing representative traces of the various experimental groups, we surprisingly observed that the application of HFS at sixteenth min of recording induces the onset of an epileptic trend only in the B-TRAINED group, which is maintained throughout the duration of the electrophysiological recording.

Figure 3b shows the PS values of Basal Synaptic Transmission (BST), before tetanic stimulation (HFS), at min 1, immediately after HFS, at min 30 and at min 60 (CTRL WBV 100.3 ± 0.8 at BST, 325.2 ± 21.9 at min 1, 205.9 ± 24.1 at min 30 and 208.1 ± 26.6 at min 60; CTRL SED 101.0 ± 0.8 at BST, 333.2 ± 23.3 at min 1, 215.9 ± 25.7 at min 30 and 201.1 ± 25.5 at min 60; A-TRAINED 101.5 ± 0.9 at BST, 206.5 ± 26.6 at min 1, 110.5 ± 25.9 at min 30 and 97.1 ± 30.3 at min 60; B-TRAINED 101.0 ± 0.9 at BST, 223.8 ± 19.6 at min 1, 172.3 ± 27.4 at min 30 and 180.3 ± 28.7 at min 60; C-TRAINED 101.4 ± 0.9 at BST, 386.8 ± 25.2 at min 1, 278.0 ± 26.9 at min 30 and 225.9 ± 29.3 at min 60).

**Figure 4:**
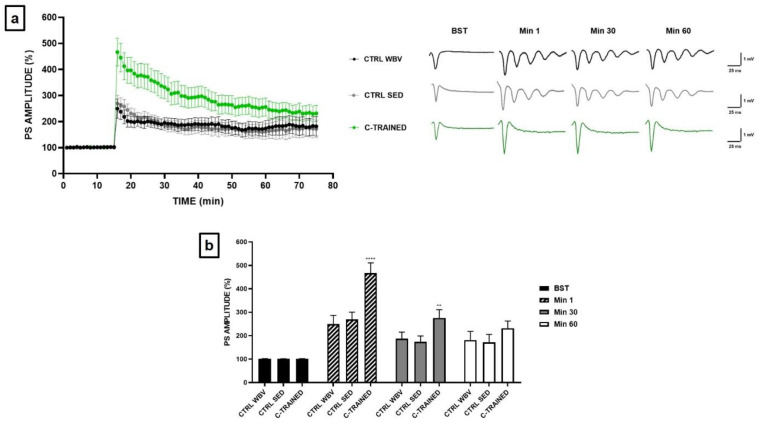
Synaptic plasticity in CA1 hippocampal subfield of old mice. (**a**) %PS amplitude as a function of time after HFS, applied at time t = 16, is shown in CTRL WBV (black line, *n* = 7), in CTRL SED (grey line, *n* = 6) and in C-TRAINED (green line, *n* = 8) mice slices. The insert shows representative recordings obtained from slices of each experimental group. The first curve of each group refers to the BST and it was recorded before the application of a high-frequency stimulation (HFS, 100 Hz for 1 s), while the other curves refer to population spikes at times 1, 30 and 60 min after the HFS. (**b**) The PS values of BST (black bar), at min 1 (diagonal pattern bar), immediately after HFS, at min 30 (grey bar) and at min 60 (white bar) from the HFS are shown for each experimental group. Bars in the plot are means ± SEM of values obtained from different slices. Note that a significant statistical difference was reported between trained and control groups at min 1 (CTRL WBV and CTRL SED vs. C-TRAINED, **** *p* < 0.0001) and at min 30 (CTRL WBV and CTRL SED vs. C-TRAINED, ** *p* < 0.01). The hippocampal slices (450 μm thick) were constantly perfused by a flow of 1.2 mL/min of ACSF and humidified gas (95% O_2_, 5% CO_2_) at 32–34 °C (pH 7.4).

Based on the results obtained for young mice, we thought to apply to the old mice only the C training protocol, which was the only one that positively modulate synaptic plasticity. Furthermore, this protocol seemed to be the least stressful and, therefore, most suitable for mice that, at this age, are particularly fragile and more susceptible to death. Figure 4a shows how in the control groups the PS amplitude was significantly reduced in the first twenty min after HFS, while PS values remained stable until the end of the electrophysiological recording. Furthermore, as shown in the insert of Figure 4b comparing the representative traces of the various experimental groups, we observed the development of an epileptic trend following HFS at the sixteenth min of recording in both control groups. Surprisingly, we observed a strong and significant effect of vibratory training in the hippocampal slices of C-TRAINED group, since the suppression of synaptic plasticity in the first min after HFS, which was present in the control groups, was completely counteracted (**** *p* < 0.0001), with stable PS values until the end of the electrophysiological recording. 

Figure 4b shows the PS values of BST, before HFS, at min 1, immediately after HFS, at min 30 and at min 60 (CTRL WBV 101.5 ± 0.8 at BST, 249.5 ± 36.9 at min 1, 187.3 ± 28.3 at min 30 and 181.7 ± 37.0 at min 60; CTRL SED 101.0 ± 0.7 at BST, 269.3 ± 31.4 at min 1, 173.3 ± 25.6 at min 30 and 171.7 ± 34.1 at min 60; C-TRAINED 101.4 ± 0.9 at BST, 466.9 ± 43.7 at min 1, 276.2 ± 34.8 at min 30 and 231.3 ± 31.4 at min 60). 

## 4. Discussion

As far as we know, this was the first experimental study designed to determine whether hippocampal synaptic plasticity could be modulating by exposure to vibratory training, applying three different protocols in terms of vibration frequency and exposure time to vibration to 4- and 24-months old mice. We first checked whether vibratory training had effects on physical parameters, such as body weight. Our results showed that all three types of training protocols induce significant weight increase in young mice. In detail, we recorded a weight increase of 49% in A-TRAINED mice and 59% both in B- and C-TRAINED mice, while in the control groups the weight increase was only 14%. These results are very satisfactory and confirm the beneficial effects of vibratory training. In fact, for the control groups, we can assert that the slight weight increase is due to the physiological development process that occurs during the growth phase. As regards the groups of trained mice, the weight increase could also depend on an increase in muscle volume determined precisely by exposure to vibratory training. In this regard, it has been shown that the application of mechanical vibrations, varying in intensity and duration, produces positive effects on bone, muscle, and joint structure, so that both tissue mass and strength are maintained at a high level, with a consequent reduction in muscle and bone loss [11]. It would also appear that vibration training generates an increase in energy metabolism and a reduction in the percentage of body fat [29]. Contrary to the results obtained for young mice, we observed a slight reduction in body weight in old mice (CTRL about 10% and C-TRAINED about 2%), which however appears to be significant only for the control groups. This result is in line with the scientific evidence present in the literature, according to which, with the aging process, the body undergoes a weight loss linked to both environmental and biological factors [30]. The C vibratory training protocol administered at this age at least prevent the reduction of mice body weight induced by physiological sarcopenia. 

Since it has been reported that exercise exerts beneficial effects on brain function [31,32,33] and that the improvement of synaptic plasticity depends on the type of training proposed [34], in the present work we have tested whether different vibratory training protocols can influence synaptic plasticity. It is known, in fact, that the synaptic changes, which are the basis of cognitive processes, depend on physiological mechanisms including LTP [35], particularly represented in the hippocampus, which is a fundamental area for memory and learning [36,37]. The effects of vibratory training on the synaptic plasticity expression have been analyzed in the CA1 region of hippocampal slices in both young and old mice. Our results have shown that in young mice only the C protocol seems to exert positive effects on synaptic plasticity, since we observed an increase in PS amplitude following HFS compared to the other experimental groups. On the contrary, the A protocol would seem to be the most stressful one, probably due to the reduced recovery time and the high vibration frequency, since we observed a significant impairment of synaptic plasticity following HFS throughout the electrophysiological recording. The B protocol appears to exert a less negative effect on synaptic plasticity than the total abrogation observed in A-TRAINED mice. In this case, in fact, PS values seems to settle on values quite like those of the control groups from the twentieth min after HFS. However, carrying out a deeper analysis and evaluating in detail the recordings obtained from the hippocampal slices of each experimental group, we surprisingly observed in young B-TRAINED mice the development of an epileptic tendency following the application of a high tetanic stimulation frequency at the sixteenth min of recording.

Our results show that vibrations of different frequencies can have opposite effects on synaptic plasticity. In this regard, it is known that vibratory perception constitutes a mechanical type of sensitivity involving mechanoreceptors, i.e., receptor structures capable of receiving vibratory signals from different parts of the body [21,23]. It has been demonstrated that for certain characteristics of frequency and amplitude of the applied vibration, these receptors generate frequencies of action potentials faithful to the frequency of the applied vibration, thus driving the activated afferents to a discharge frequency identical to that of stimulation [21,22]. Therefore, we hypothesized that vibration frequency, depending on the protocol used, can modify the analysis of the proprioceptive information, while the persistence of the effects could suggest the actual induction of plastic modifications of the proprioceptive circuitry.

Based on the obtained results, we thought of training old mice with only the C protocol, which would seem the least stressful and, therefore, the most suitable for them, which, at this age, are particularly fragile and more susceptible to death. It is well known that aging is a biological process associated with physiological cognitive decline, as it can impair quality of life and cause deficits in declarative and working memory, spatial learning and attention [26]. Although increased physical activity has been proposed as an effective therapeutic strategy to reduce the decline in muscle and cognitive function, to date most of the underlying molecular mechanisms are still unclear. In this regard, it has been shown that during the aging process exercise exerts beneficial effects in several brain functions, activating neurogenesis and delaying neurodegenerative processes [33]. Most recently, it has been reported that exercise also has positive effects on synaptic plasticity [32]. In accordance with this evidence, we have surprisingly observed a strong and significant effect of vibratory training on synaptic plasticity in the trained mice group, since the suppression of synaptic plasticity in the first min after HFS, which was present in sedentary mice, was completely counteracted. Regarding the control groups, we obtained results very similar to those of the group of young B-TRAINED mice. In fact, not only extracellular field potential recordings provided overlapping results, but also in 24-month controls mice we found the development of a similar epileptic tendency after the application of a high frequency tetanic stimulation at the sixteenth min of recording. 

The problem of how temporal lobe epilepsy affects brain structure and cognition during lifespan remains a topic of interest and concern. To date, it is known that brain structures related to memory are directly involved in the epileptic process: in fact, memory disorders and deficits are common in patients with epilepsy, especially for people suffering by Temporal Lobe Epilepsy (TLE). In recent decades, epidemiological studies have shown that the highest incidence of epileptic seizures is found in the elderly [38]. In fact, the incidence of risk factors for epilepsy increases with advancing age, and the aging process of neurons itself is considered a risk factor for the development of this pathology [39]. Based on this evidence, we hypothesized that the epileptic trend found in old sedentary mice is linked precisely to the cognitive deterioration associated with the advanced age of the animals, and that vibratory training can reduce this effect. Therefore, vibratory training seems to represent an important protective and preventive factor, since by protecting brain health, it is possible to prevent and/or delay the development of age-related cognitive impairment. As regards, instead, the young B-TRAINED mice, the epileptic tendency would seem to be related precisely to the type of protocol used which, so organized in terms of vibration frequency and exposure time to vibration, could represent a source of considerable stress and damage to the hippocampus and other brain structures related to memory and directly involved in the epileptic process.

## 5. Conclusions

The results obtained from this experimental study suggest that vibratory training exerts positive effects on cognitive processes provided that certain principles, such as the vibration frequency and the exposure time to vibration, are respected, in order to plan the right parameters quantitative and qualitative and appropriate recovery periods. Furthermore, we can speculate that any alteration in neuronal transmission from the vibratory sensors to the somatosensory cortex could influence vibratory sensations, with specific effects at the hippocampal level. Specifically, depending on the vibration frequency used, a change in the analysis of proprioceptive information may occur, while the persistence of the effects could suggest the actual induction of plastic changes in the proprioceptive circuit. Furthermore, we suggest that the effects of vibration training on cognitive processes may vary with age, which in turn appears to be closely related to synaptic plasticity. Thus, vibration training may be considered an important factor in protecting and/or preventing the development of age-related cognitive impairments. Further studies will aim to understand brain responses to various types of vibratory training protocol and explore the underlying mechanisms. 

## Data Availability

The data presented in this study are available on request from the corresponding author.
